# Aetiologies and factors associated with mortality among HIV-infected patients at Taraba State Specialist Hospital, Jalingo, Nigeria

**DOI:** 10.1186/1758-2652-13-S4-P221

**Published:** 2010-11-08

**Authors:** DR Dashe

**Affiliations:** 1 Taraba state specialist Hospital, Jalingo, HIV/AIDS, Jalingo, Nigeria

## Background

Despite improved access to life-saving antiretroviral drugs, HIV associated mortality remains high in most settings in sub-Saharan Africa. Several studies have shown remarkable temporal distribution of these mortality figures, with a disproportionately higher number of patients dying within the first few months of commencing HAART.

## Purpose of the study

To identify the aetiologies and factors associated with the deaths of HIV-infected persons enrolled into the HIV care program at our hospital.

## Methods

The study setting is a 230- bed Hospital located in the north eastern region of Nigeria, with HIV prevalence of 5.2%. HIV services are supported by Management Sciences for Health through USAID. A cross sectional study was conducted. The case files of all recorded deaths were retrieved from the medical records department and information on age, sex, date of enrolment, date of commencement of anti-retroviral drugs, duration of HAART, W.H.O clinical stage, CD4 cell count and aetiologies/factors associated with the mortalities were extracted and analyzed.

## Summary of results

A total of 1510 HIV positive patients were enrolled in our centre (63%F, 47%M) with 758 on HAART. 80 patients (5.2%) had died as at the time of the review. 63% of mortality cases were in patients<40yrs old and 82% presented with WHO stage 3 or 4 disease. The aetiology of death was TB in 40%, suspected TB in 16.25%, HIV wasting syndrome in 8.75%, septic abortion in 1.25% and HBV in 1.25%. In 30% of the cases however, the cause was not ascertained before death. 52% of mortality cases occurred before HAART commencement, 35% occurred within the first 3 months of HAART initiation. 60% of deaths occurred in patients with CD4+T-cell counts<200cells/uL

## Conclusions

Advanced HIV disease, severe immunodeficiency, TB co-morbidity and possibly delay in starting HAART were found to be associated with increased mortality in our centre. Thus, strategies to reduce mortality must include earlier diagnosis of HIV infection and timely initiation of HAART.

